# Telocytes in the mouse testicular interstitium: implications of G-protein-coupled estrogen receptor (GPER) and estrogen-related receptor (ERR) in the regulation of mouse testicular interstitial cells

**DOI:** 10.1007/s00709-018-1305-2

**Published:** 2018-09-05

**Authors:** Piotr Pawlicki, Anna Hejmej, Agnieszka Milon, Krzysztof Lustofin, Bartosz J. Płachno, Waclaw Tworzydlo, Ewelina Gorowska-Wojtowicz, Bernadetta Pawlicka, Malgorzata Kotula-Balak, Barbara Bilinska

**Affiliations:** 1 Department of Endocrinology, Institute of Zoology and Biomedical Research, Jagiellonian University in Kraków, Gronostajowa 9, 30-387 Krakow, Poland; 2 Department of Plant Cytology and Embryology, Institute of Botany, Jagiellonian University in Kraków, Gronostajowa 9, 30-387 Krakow, Poland; 3 Department of Developmental Biology and Invertebrate Morphology, Institute of Zoology and Biomedical Research, Jagiellonian University in Kraków, Gronostajowa 9, 30-387 Krakow, Poland; 4 Department of Genetics and Evolution, Institute of Zoology and Biomedical Research, Jagiellonian University in Kraków, Gronostajowa 9, 30-387 Krakow, Poland

**Keywords:** Cell biology, G estrogen receptor, Estrogen-related receptor, Mouse, Telocytes, Testis, Ultrastructure

## Abstract

Telocytes (TCs), a novel type of interstitial cells, are involved in tissue homeostasis maintenance. This study aimed to investigate TC presence in the interstitium of mouse testis. Additionally, inactivation of the G-coupled membrane estrogen receptor (GPER) in the testis was performed to obtain insight into TC function, regulation, and interaction with other interstitial cells. Mice were injected with a GPER antagonist (G-15; 50 μg/kg bw), and the GPER-signaling effect on TC distribution, ultrastructure, and function, as well as the interstitial tissue interaction of GPER with estrogen-related receptors (ERRs), was examined. Microscopic observations of TC morphology were performed with the use of scanning and transmission electron microscopes. Telocyte functional markers (CD34; c-kit; platelet-derived growth factor receptors α and β, PDGFRα and β; vascular endothelial growth factor, VEGF; and vimentin) were analyzed by immunohistochemistry/immunofluorescence and Western blot. mRNA expression of CD34 as well as ERR α, β, and γ was measured by qRT-PCR. Relaxin and Ca^2+^ concentrations were analyzed by immunoenzymatic and colorimetric assays, respectively. For the first time, we reveal the presence of TCs in the interstitium together with the peritubular area of mouse testis. Telocytes were characterized by specific features such as a small cell body and extremely long prolongations, constituting a three-dimensional network mainly around the interstitial cells. Expression of all TC protein markers was confirmed. Based on scanning electron microscopic observation in GPER-blocked testis, groups of TCs were frequently seen. No changes were found in TC ultrastructure in GPER-blocked testis when compared to the control. However, tendency to TC number change (increase) after the blockage was observed. Concomitantly, no changes in mRNA CD34 expression and increase in ERR expression were detected in GPER-blocked testes. In addition, Ca^2+^ was unchanged; however, an increase in relaxin concentration was observed. Telocytes are an important component of the mouse testicular interstitium, possibly taking part in maintaining its microenvironment as well as contractile and secretory functions (via themselves or via controlling of other interstitial cells). These cells should be considered a unique and useful target cell type for the prevention and treatment of testicular interstitial tissue disorders based on estrogen-signaling disturbances.

## Introduction

Telocytes have been previously described by Popescu et al. (2005) in human pancreas, fallopian tube, and cardiac, digestive, and reproductive systems where they were named interstitial Cajal-like cells. Based on their extremely long prolongations (telopodes), telocytes (TCs) are easily distinguished from other interstitial tissue cells (Popescu and Faussone-Pellegrini 2010). Telopodes are hundreds of micrometers long and extremely thin (between 0.05 and 0.2 μm), making up a succession of thin, fibrillar segments (podomers) and dilated, cistern-like regions (podoms) (Cretoiu and Popescu 2014). Podoms accommodate functional units consisting of caveolae, mitochondria, and endoplasmic reticulum, possibly involved in calcium ion (Ca^2+^) uptake and release (Cretoiu et al. 2012). These cells are interconnected by homo- and heterocellular junctions to form three-dimensional networks within the interstitial tissue (Cretoiu and Popescu 2014). In TC ultrastructure, large numbers of mitochondria, an abundance of endoplasmic reticulum and lipid droplets, and distinct sets of membrane channels are observed. Recent studies report the involvement of TCs in processes occurring at the cellular level: organizational regulation and activity of the extracellular matrix, structural support, formation of microenvironments, intercellular communication, neurotransmission, immunomodulation and immune surveillance, cell survival and apoptosis, and control of other cell types in the interstitium (Díaz-Flores et al. 2016). Telocytes are widely distributed in the interstitium of various organs as well as in serous membranes of vertebrates (fish, reptiles, birds, and mammals, including humans) (Popescu et al. 2010; Mostafa et al. 2010; Hinescu et al. 2011; Popescu 2011; Sanders et al. 2014; Yang et al. 2014).

In tissue pathology, variations in TC number have been demonstrated in both experimental and clinical studies. For example, in mice after hepatic resection, liver tissue regeneration was observed. Telocytes affected hepatocyte proliferation and/or hepatic stem cell differentiation via intercellular junctions and ectovesicles (Wang et al. 2014). Reduction in TC number, correlating with fibrosis, was noted in the gastrointestinal tract of patients with Crohn’s disease (Milia et al. 2013). The loss of TCs was revealed in the aging heart, while in myocardial fibrotic areas of myocardial infarction and systemic sclerosis, TCs were almost completely absent, indicating their impaired function (Popescu et al. 2015). Alternatively, in cell fibrosis, TCs promoted cell hypertrophy as was demonstrated in exercise-induced cardiac cells (Xiao et al. 2016).

Minimal data is available on the localization and role of TCs in the male reproductive system. Mokhtar et al. (2016) revealed TCs in seminal vesicles of the Soay ram. In the prostate, the involvement of TCs in tissue organization during postnatal development has been demonstrated (Sanches et al. 2017). Telocytes were found in human testis, particularly in men with prostate cancer (based on c-kit immunostaining) and those with nonobstructive azoospermia (Rodríguez et al. 2008; Hasirci et al. 2017). The latter study confirmed that the number and distribution of TCs may affect spermatogenesis. The number of TCs was higher in patients with nonobstructive azoospermia than in those with obstructive azoospermia (Hasirci et al. 2017). Yang et al. (2015) reported the presence of TCs in the testis of the Chinese softshell turtle (*Pelodiscus sinensis*)*.*

In the testis, the interstitium is located in between seminiferous tubules. This tissue is composed of loose connective tissue with blood and lymph vessels, macrophages, fibroblasts, mast cells, lymphocytes, and Leydig cells, which are the main component (Christensen 1975). The peritubular area, with a layer of smooth muscle cells (peritubular-myoid cells), directly delimits seminiferous tubules from the interstitium. Multiple and multilevel interactions of molecules secreted by testicular cells, or transported to the testis from other tissues, are responsible for maintaining the local microenvironment that is crucial for spermatogenic and steroidogenic functions (Skinner et al. 1991).

Estrogen signaling in the male reproductive system is important for proper fertility (Hess 2003). Responses to estrogens, both genomic and rapid signaling, are initiated by nuclear receptors: estrogen receptors (ERs), their splice-variants, membrane G-coupled estrogen receptor (GPER), and estrogen-related receptors (ERRs) (Bjornstrom and Sjoberg 2005; Vrtačnik et al. 2014). While ERRs show a high degree of DNA sequence homology to ERs, and function alongside the estrogen mechanism of action (Huppunen and Aarnisalo 2004), GPER has different biochemical and molecular characteristics. This seven-transmembrane domain receptor is associated with G proteins, thus playing a fundamental role in coupling external stimuli with cytoplasmic and nuclear targets after activation, and is dependent on cooperation with growth factor receptors (Vögler et al. 2008). Indeed, due to GPER localization, it is an initial sensor of exogenous estrogen action.

Currently, multiple groups have shown the expression of novel receptors: GPER and ERR in rodent Leydig cells both in vivo and in vitro (Chimento et al. 2014; Vaucher et al. 2014; Pardyak et al. 2016; Zarzycka et al. 2016; Park et al. 2017; Pawlicki et al. 2017). The interaction of GPER with intratesticular estrogen levels was reported in male bank voles with normal and physiologically decreased estrogen levels as well as in male mice of various ages (Zarzycka et al. 2016; Kotula-Balak et al. 2018). Also, ERR regulation via serum hormonal factors, as well as phytoestrogens and synthetic hormonally active compounds, was demonstrated in various tissues (Vanacker et al. 1999; Roshan-Moniri et al. 2014; Pardyak et al. 2016; Milon et al. 2017). Moreover, the implication of both GPER and ERR action in testicular steroidogenic cell function was shown (Vaucher et al. 2014; Park et al. 2017). Despite the fact that the role of lipid droplets in TCs is not recognized, their function was found to be controlled by Ca^2+^ signaling (Radu et al. 2017).

This study aimed to explore the presence of TCs in the mouse testicular interstitium. In addition, we focused on interstitium function as a result of GPER and ERR signaling as well as the possible involvement of TCs in maintaining proper interstitial cell histology and function.

## Materials and methods

### Animals and treatments

Male mice (C57BL/6), 3 months old (*n* = 10), were obtained from the Department of Genetics and Evolution, Institute of Zoology and Biomedical Research, Jagiellonian University, Kraków. Animals were maintained on 12 h dark-light (250 lx at cage level) cycle with stable temperature condition (22 °C), relative humidity of 55 ± 5%, and free access to water and standard pelleted diet (LSM diet, Agropol, Motycz, Poland). Animals were killed by cervical dislocation. The use of the animals was approved by the National Commission of Bioethics at the Jagiellonian University in Krakow, Poland (No. 151/2015).

Mice were allotted into experimental groups (each group including five animals) and control (Cont.) and treated with a selective GPER receptor antagonist [(3aS*,4R*,9bR*)-4-(6-bromo-1,3-benzodioxol-5-yl)-3a,4,5,9b-3*H*-cyclopenta[*c*]quinolone; G-15] (Tocris Bioscience, Bristol, UK). G-15 was dissolved in DMSO and the stock solutions were kept at − 20 °C. Animals from the experimental groups were injected subcutaneously with freshly prepared solutions of G-15 (50 μg/kg bw) in phosphate-buffered saline (six doses each dose injected every other day). Mice from control groups received vehicle only. Dose, frequency, and time of G-15 administration were based on our previous study (Kotula-Balak et al. 2018). Both testes of each individual of control and G-15-treated mice were surgically removed and were cut into small fragments. For histological appearance and immunohistochemistry, tissue samples were fixed in 10% formalin and embedded in paraplast, or small pieces of the testicular tissue were immediately fixed in formaldehyde and glutaraldehyde (see “Materials and methods”: “Cell topography—scanning electron microscope (SEM)” and “Cell ultrastructure—transmission electron microscope (TEM)” sections, respectively) for transmission microscopy analysis or frozen in liquid nitrogen and stored at − 80 °C for RNA isolation and determination of steroid hormones.

### Cell topography—scanning electron microscope (SEM)

Control and G-15-treated testes were fixed in a mixture of 2.5% formaldehyde with 2.5% glutaraldehyde in a 0.05-M cacodylate buffer (Sigma; pH 7.2) for several days, washed three times in a 0.1-M sodium cacodylate buffer, and later dehydrated and subjected to critical-point drying. They were then sputter-coated with gold and examined at an accelerating voltage of 20 or 10 kV using a Hitachi S-4700 scanning electron microscope (Hitachi, Tokyo, Japan).

### Cell ultrastructure—transmission electron microscope (TEM)

Control and G-15-treated fragments of testes were immersed in ice-cold prefixative containing 2% formaldehyde and 2.5% glutaraldehyde in 0.1 M phosphate buffer, pH 7.3 overnight at 4 °C. The tissues were then rinsed and postfixed in a mixture of 2% osmium tetroxide and 0.8% potassium ferrocyanide in the same buffer for 30 min at 4 °C (Russell and Burguet 1977; McDonald 1984). The material was embedded in Glycid Ether 100 resin (Serva, Heidelberg, Germany). Semithin sections (0.7 μm thick) were stained with 1% methylene blue and examined under a Leica DMR (Wetzlar, Germany) microscope. Prior to embedding, small (3–5 mm) pieces of testicular tissue were carefully oriented in the mold to obtain accurate cross sections of the interstitial tissues and tubules. Ultrathin sections (80 nm thick) were contrasted with uranyl acetate and lead citrate and analyzed with a JEOL 2100 HT (Japan) TEM.

### RNA isolation and reverse transcription

Total RNA was extracted from control and G-15-treated mouse testes using TRIzol® reagent (Life Technologies, Gaithersburg, MD, USA) according to the manufacturer’s instructions. The yield and quality of the RNA were assessed using a NanoDrop ND2000 Spectrophotometer (Thermo Scientific, Wilmington, DE, USA). Samples with a 260/280 ratio of 1.95 or greater and a 260/230 ratio of 2.0 or greater were used for analysis. Total cDNA was prepared using High-Capacity cDNA Reverse Transcription Kit (Applied Biosystems, Carlsbad, CA, USA) according to the manufacturer’s instructions.

The purified total RNA was used to generate total cDNA. A volume equivalent to 1 μg of total RNA was reverse transcribed using the High-Capacity cDNA Reverse Transcription Kit (Applied Biosystems, Carlsbad, CA, USA) according to the manufacturer’s instructions. Total cDNA was prepared in a 20-μL volume using a random primer, dNTP mix, RNase inhibitor, and reverse transcriptase (RT). Parallel reactions for each RNA sample were run in the absence of RT to assess genomic DNA contamination. RNase-free water was added in place of the RT product.

### Real-time quantitative RT-PCR

Real-time RT-PCR was performed using the StepOne Real-Time PCR system (Applied Biosystems) and optimized standard conditions as described previously by Kotula-Balak et al. (2013, 2018). Based on the gene sequences in Ensembl database, primer sets were designed using Primer3 software (Table 1). Selected primers were synthesized by the Institute of Biochemistry and Biophysics, Polish Academy of Sciences (Warsaw, Poland).

**Table 1:** Sequences of forward and reverse primers

Genes	Primers (5′–3′)	Product size (bp)	Annealing temperature (°C)	Cycles
CD34	5′-TAGCTCTCTGCCTGATGAGTCTGCTG-3′ 5′-CTGAGATGGCTGGTGTGGTCTTACTG-3′	234	61.1	40
ERRα	5′-GCCTCTACCCAAACCTCTCT-3′ 5′-AGCCAT CCCTCCTTCGCACA-3′	234	60	40
ERRβ	5′-GAGCCATCTTTACCGCTGGA-3′ 5′-CAGCTTGTCAACAGGCAGTG-3′	239	60	40
ERRγ	5′-CTTGTAATGGGGTTGCCTC-3′ 5′-TATCACCTTCTGCCGACCT-3′	222	62	40
β-Actin	5′-AAGTACCCCATTGAACACGG-3′ 5′-ATCACAATGCCAGTGGTACG-3′	274	52	40

*ERRα*, estrogen-related receptor alpha; *ERRβ*, estrogen-related receptor beta; *ERRγ*, estrogen-related receptor gamma

To calculate the amplification efficiency, serial cDNA dilution curves were produced for all genes (Pfaffl 2001). A graph of threshold cycle (Ct) versus log10 relative copy number of the sample from a dilution series was produced. The slope of the curve was used to determine the amplification efficiency: %E = (10^–1/slope^ − 1) × 100. All PCR assays displayed efficiency between 94 and 104%.

Detection of amplification products for CD34, ERRα, ERRβ, and ERRγ, and for the reference gene β-actin, was performed with 10 ng cDNA, 0.5 μM primers, and SYBR Green master mix (Applied Biosystems) in a final volume of 20 μL. Amplifications were performed as follows: 55 °C for 2 min, 94 °C for 10 min, followed by annealing temperature for 30 s (Table 1) and 45 s 72 °C to determine the cycle threshold (Ct) for quantitative measurement as described previously (Kotula-Balak et al. 2013). To confirm amplification specificity, the PCR products from each primer pair were subjected to melting curve analysis and subsequent agarose gel electrophoresis (not shown). In all real-time RT-PCR reactions, a negative control corresponding to RT reaction without the reverse transcriptase enzyme and a blank sample were carried out. All PCR products stained with Midori Green Stain (Nippon Genetics Europe GmbH, Düren, Germany) were run on agarose gels. Images were captured using a Bio-Rad Gel Doc XR System (Bio-Rad Laboratories, Hercules, CA, USA) (not shown). CD34, ERRα, ERRβ, and ERRγ mRNA expressions were normalized to the β-actin mRNA (tested with other references genes: GAPDH and Tuba1α in a pilot study) (relative quantification, RQ = 1) with the use of the 2^−ΔΔCt^ method, as previously described by Livak and Schmittgen (2001).

Three independent experiments were performed, each in triplicate with tissues prepared from different animals.

### Western blot

Lysates of testes (of the control and GPER-blocked) were obtained by sample homogenization and sonication with a cold Tris/EDTA buffer (50 mM Tris, 1 mM EDTA, pH 7.5), supplemented with broad-spectrum protease inhibitors (Sigma-Aldrich). The protein concentration was estimated by the Bio-Rad DC Protein Assay Kit with BSA as standard (Bio-Rad Labs, GmbH, München, Germany). Equal amounts of protein were resolved by SDS-PAGE under reducing conditions, transferred to polyvinylidene difluoride membranes (Merck Millipore, Darmstadt, Germany), and analyzed by Western blotting with antibodies listed in Table 2. The presence of the primary antibody was revealed with horseradish peroxidase-conjugated secondary antibodies diluted 1:3000 (Vector Lab., Burlingame, CA, USA) and visualized with an enhanced chemiluminescence detection system as previously described (Zarzycka et al. 2016). All immunoblots were stripped with stripping buffer containing 62.5 mM Tris-HCl, 100 mM 2-mercaptoethanol, and 2% SDS (*w*/*v*; pH 6.7) at 50 °C for 30 min and incubated in antibody against β-actin (loading control). Three independent experiments were performed, each in triplicate with tissues prepared from different animals. To obtain quantitative results, the bands (representing each data point) were densitometrically scanned using the public domain ImageJ software (National Institutes of Health, Bethesda, MD, USA) (Smolen 1990). The data obtained for each protein were normalized against its corresponding actin and expressed as relative intensity. Results of 10 separate measurements were expressed as mean ± SD.

**Table 2:** Primary antibodies used for immunohistochemistry and Western blotting

Antibody	Host species	Vendor	Dilution
CD34	Rabbit	Abcam Cat. no. ab81289	1:200 (IHC) 1:500 (WB)
c-kit	Rabbit	Thermo Fisher Cat. no. #34-8800	1:1000 (IHC) 1:500 (WB)
PDGFRα	Rabbit	Cell Signaling Technology Cat. no. #3174	1:500 (IHC) 1:500 (WB)
PDGFRβ	Mouse	Abcam Cat. no. ab69506	1:300 (IHC) 1:500 (WB)
VEGF	Rabbit	Merck Cat. no. #07-1420	1:300 (IHC) 1:500 (WB)
Vimentin	Rabbit	Cell Signaling Technology Cat. no. #5741	1:200 (IHC) 1:500 (WB)
β-Actin	Mouse	Sigma-Aldrich Cat. no. A2228	1:3000 (WB)

*c-kit*, tyrosine-protein kinase kit, *PDGFRα*, platelet-derived growth factor receptor α; *PDGFRβ*, platelet-derived growth factor receptor β; *VEGF*, vascular endothelial growth factor

### Immunohistochemistry and immunofluorescence

To optimize immunohistochemical staining testicular sections (4 μm thin), both control and G-15-treated mice were immersed in 10 mM citrate buffer (pH 6.0) and heated in a microwave oven (2 × 5 min, 700 W). Thereafter, sections were immersed sequentially in H_2_O_2_ (3%; *v*/*v*) for 10 min and normal goat or horse serum (5%; *v*/*v*) for 30 min which were used as blocking solutions. After overnight incubation at 4 °C with primary antibodies listed in Table 2, the next respective biotinylated antibodies (anti-rabbit, anti-goat, and anti-mouse IgGs; 1: 400; Vector, Burlingame CA, USA) and avidin-biotinylated horseradish peroxidase complex (ABC/HRP; 1:100; Dako, Glostrup, Denmark) were applied in succession. Bound antibody was visualized with 3,3′-diaminobenzidine (DAB) (0.05%; *v*/*v*; Sigma-Aldrich) as a chromogenic substrate. Control sections included omission of primary antibody and substitution by irrelevant IgG. Thereafter, sections were washed and were slightly counterstained with Mayer’s hematoxylin and mounted using DPX mounting media (Sigma-Aldrich).

To count TC number per testicular section, the volume of CD34-positive cells per section was determined by a point-counting method using a graticule with 121 points (according to Sharpe et al. 2000 with modifications). Serial testicular sections (three to five) from each of the animals (control and G-15-treated mice) were examined. Applying a systematic sampling pattern from a random starting point, approx. 60 fields were counted. Results were expressed as mean number per testicular section.

Fluorescence labeling for F-actin was performed on testicular sections fixed in absolute methanol for 7 min followed by acetone for 4 min both at − 20 °C, respectively. Next, sections were rinsed in TBS containing 0.1% Triton X-100. Thereafter, cells were incubated with rhodamine-conjugated phalloidin (cat. no. R415, Invitrogen Molecular Probes) that recognizes F-actin for 30 min in a dark chamber for 30 min in a humidified chamber. After this step, cells were carefully rinsed with TBS. Fluorescent staining was protected from light and cells were mounted with Vectashield mounting medium (Vector Labs) with 4′,6-diamidino-2-phenylindole (DAPI) and next examined with epifluorescence microscope Leica DMR (Leica Microsystems) equipped with appropriate filters. Experiments were repeated three times.

Counting of F-actin-positive cells was performed on 10 randomly chosen microscopic high-power fields (hpf; ×40) of the testicular sections according to Manetti et al. (2013). Total fluorescence (a.u.) of F-actin was measured with the use of ImageJ software (NIH, Bethesda, USA) according to Smolen (1990). Briefly, to calculate total fluorescence per region, mean values for interstitial tissue areas in serial sections were averaged including the background reading with the use of NIS-Elements software and expressed as total fluorescence (a.u).

### Relaxin concentration

Relaxin concentration was measured in (100 μL) lysates of control and G-15-treated testes with the use of mouse relaxin 1 ELISA Kit (cat. no. ab213885; Abcam) according to the manufacturer’s protocol. The biological sensitivity of an assay was < 10 pg/mL. For determination of optical density, a spectrophotometer (Labtech LT-4000MS; Labtech International Ltd., Uckfield, UK) with Manta PC analysis software set to 450 nm was used.

Concentrations of relaxin in G-15-treated testes were compared with the control. Relaxin concentration was calculated as picograms per milliliter.

### Determination of Ca^2+^ concentrations

Control and G-15 testes homogenates were sonicated for 60 s on ice and centrifuged at 10,000*g* for 15 min. Ca^2+^ was estimated using Arsenazo III (Sigma-Aldrich, St. Louis, MO, USA) according to the modified method by Michaylova and Ilkova (1971). The intensity of the purple complex formed with the reagent was read at 600 nm in a spectrophotometer (Labtech LT-4000MS; Labtech International Ltd., Uckfield, UK) with Manta PC analysis software. The proteins were estimated by the modified Lowry’s method (Lowry et al. 1951). Concentrations of Ca^2+^ in G-15-treated testes were compared with the control. The Ca^2+^ concentrations were calculated as micrograms per milliliter.

### Statistical analysis

Each variable was tested by using the Shapiro-Wilk *W* test for normality. Homogeneity of variance was assessed with Levene’s test. Since the distribution of the variables was normal and the values were homogeneous in variance, all statistical analyses were performed using one-way analysis of variance (ANOVA) followed by Tukey’s post hoc comparison test to determine which values differed significantly from controls. The analysis was made using Statistica software (StatSoft, Tulsa, OK, USA). Data were presented as mean ± SD. Data were considered statistically significant at *p* < 0.05. All the experimental measurements were performed in triplicate.

## Results

### Presence of telocytes in mouse testis—SEM, TEM, and immunohistochemical and fluorescence analyses: effect of GPER blockage

**Fig. 1:**
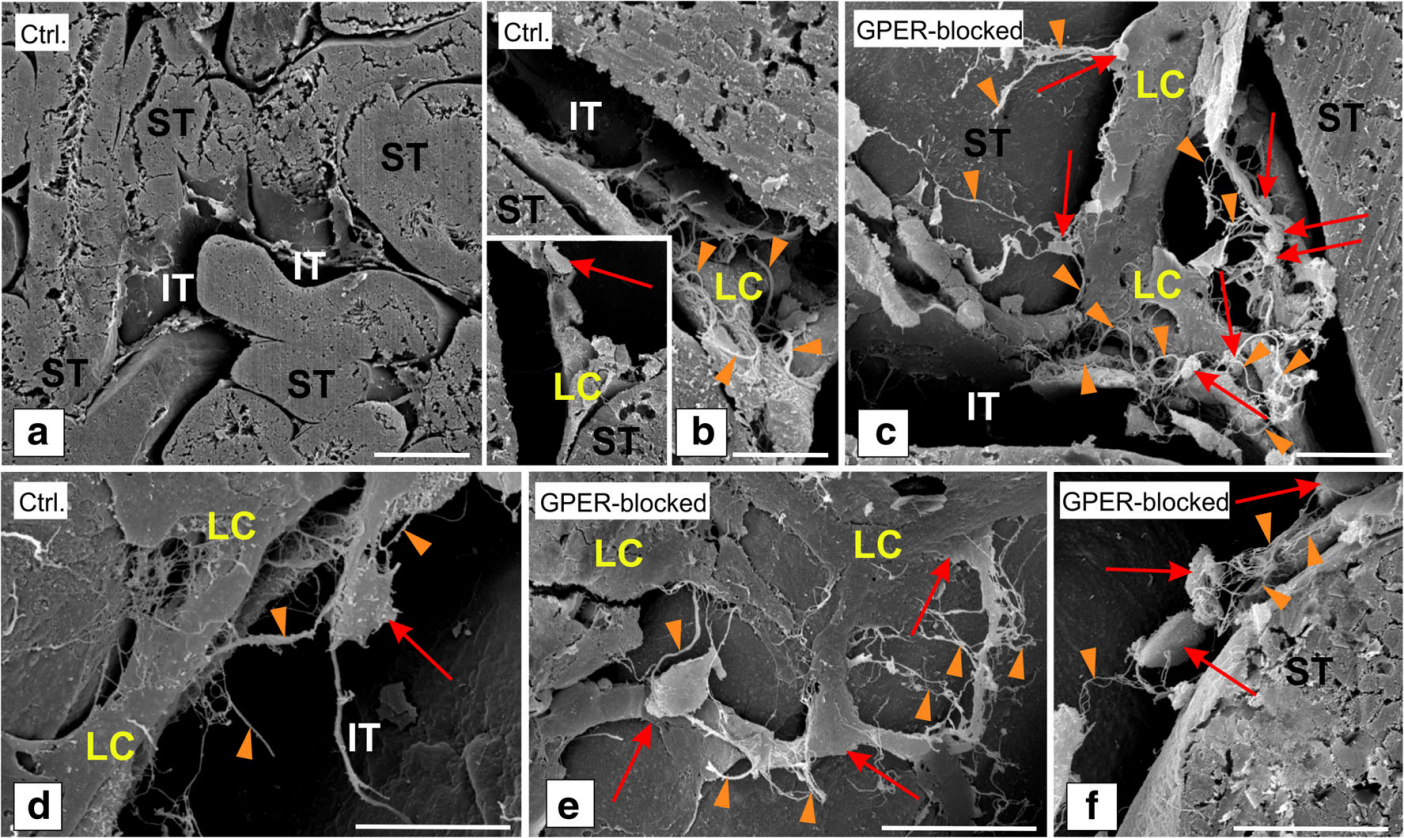
Presence of telocytes in mouse testis—SEM analysis. Effect of GPER blockage. Representative microphotographs of sections of control (**a**, **b** and insert, **d**) and GPER-blocked (**c**, **e**, and **f**) coated with gold. Bars represent 1 μm. Analysis was performed on three testicular fragments from at least three animals of each experimental group. TCs are marked with red arrows, while their long and thin protrusions (telopodes) with orange arrowheads. Note increased number of TCs in GPER-blocked testes. IT, interstitial tissue; ST, seminiferous tubules; LC, Leydig cells

In the testicular tissue, SEM analysis was utilized for observation of general interstitial cell topography. Only testis fragments with highly visible and untouched seminiferous tubules were used for analysis (Fig. 1a). The results revealed the presence of TCs in the testis interstitium between seminiferous tubules (Fig. 1b–f). In the control and GPER-blocked testes, TCs were present in both interstitial and peritubular areas and recognized by pale, small round body and very long, thin cellular prolongations (Fig. 1b–f and insert at b). Telocytes were located in close proximity to Leydig cells. The latter cells were recognized by a large polygonal body and short, wide pseudopodia located in groups where single cells tightly adhered to each other (Fig. 1b–e). In addition, TCs were present in between and/or on peritubular cells surrounding the seminiferous tubule’s basement membrane (Fig. 1f). Telocytes were found to be spatially distributed and composed a net-like structure enclosing the interstitial space containing Leydig cells (Fig. 1b–f). On the body surface of TC body, little to no very short and thin cell processes were observed (Fig. 1d). The density of TCs was different by region, and they appeared either singularly or in small groups. More frequently, groups of TCs were observed in the interstitium of GPER-blocked testes when compared to the control (Fig. 1b–d).

**Fig. 2:**
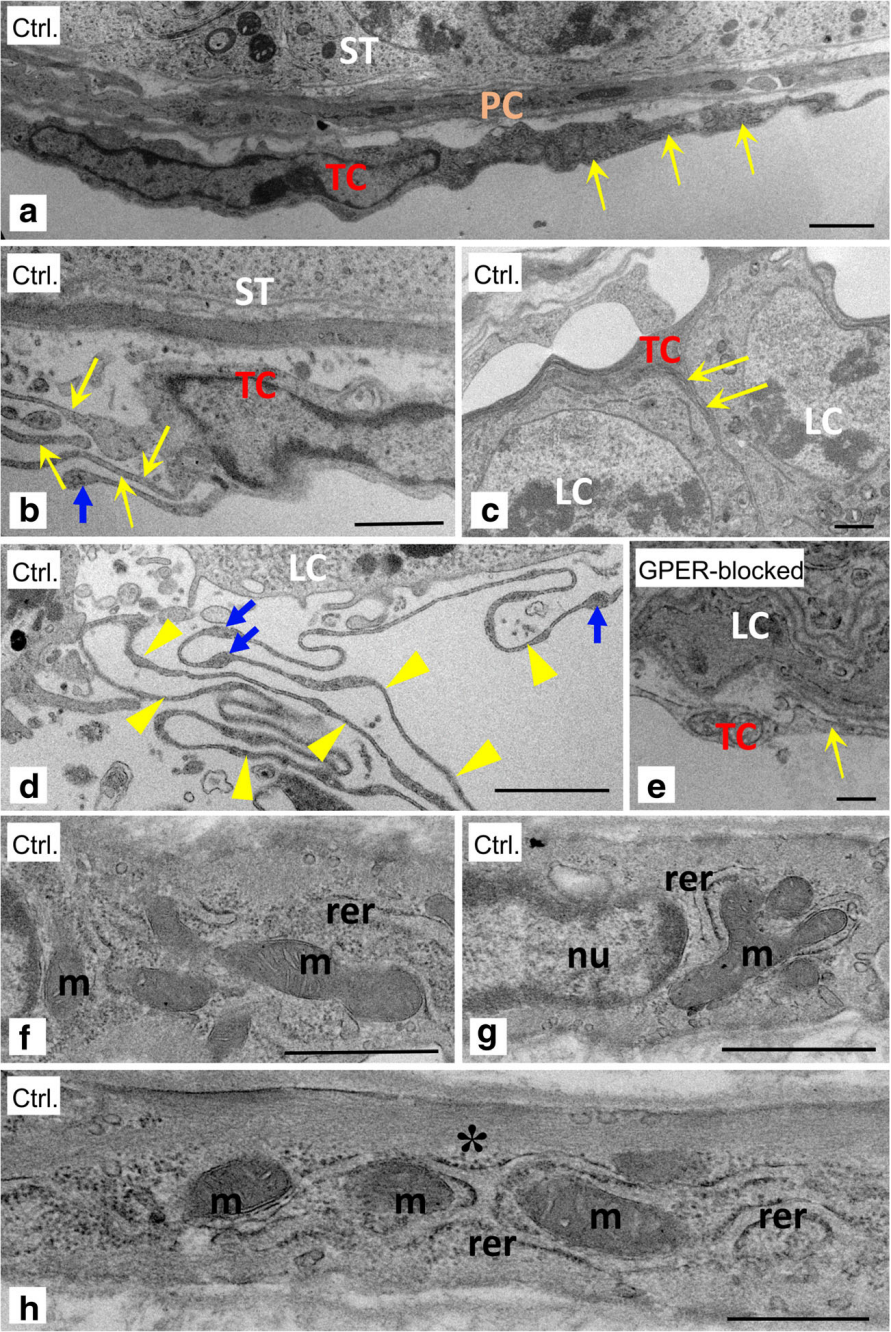
Presence of telocytes in mouse testis—TEM analysis. Effect of GPER blockage. Representative microphotographs of ultrathin sections of TCs from control (**a**–**d** and **f**–**h**) and GPER-blocked mouse testes (**e**). Bars represent 1 μm. Each testicular sample in epoxy resin block was cut for at least three ultrathin sections that were analyzed. Analysis was performed on testicular blocks from at least three animals of each experimental group. ST, seminiferous tubules; TC, telocyte; LC, Leydig cells; m, mitochondria, PC, peritubular cell; asterisk—cortical filaments; rer—elements of endoplasmic reticulum. Note long protrusions (telopodes) of the TCs (arrows). Sometimes the TCs send several protrusions toward one direction (yellow arrows in **b**). In some cases, the telopodes intertwine with one another and form characteristic labyrinths (yellow arrowheads in **d**). Note dilated fragments of the telopodes that form podomer-like structures (blue arrows in **b**). The TCs that are located in close vicinity to Leydig cells very often send protrusions that penetrate in-between adjacent Leydig cells (arrows in **c**)

Concomitantly, TCs in the control and GPER-blocked testes were analyzed by TEM (Fig. 2). Analyses of serial sections revealed the presence of TCs in both peritubular and interstitial testis compartments (Fig. 2). TCs of both localizations had a similar appearance with a relatively small, rounded cell body and extremely elongated, thin pseudopodia. Nearly the entire cell body was filled with a slightly elongated nucleus surrounded by a small rim of cytoplasm. Well-developed elements of rough endoplasmic reticulum and numerous elongated and branched mitochondria were also observed (Fig. 2a, b, f, g).

The most characteristic feature of the TCs was very long and thin cell protrusions (telopodes) that formed podom-like dilated structures (Fig. 2b, d). Occasionally, the single TC sent several elongated protrusions (Fig. 2b), but most TCs possessed a few remarkably long telopodes. The telopode cytoplasm contained mitochondria that were linearly arranged one by one (Fig. 2h). In the cortical regions of the telopode, numerous tightly packed filamentous structures were present (Fig. 2h, asterisk). Based on their structure and size, we believe the filaments represent F-actin microfilaments (Fig. 2h). In TCs surrounding the seminiferous tubule, the telopodes ran parallel to peritubular cells (Fig. 2a). In TCs that were located near Leydig cells, the long telopodes embraced Leydig cells and could be seen penetrating between adjacent Leydig cells (Fig. 2c). No characteristic changes in the ultrastructure of TCs were observed between the control and GPER-blocked testes.

**Fig. 3:**
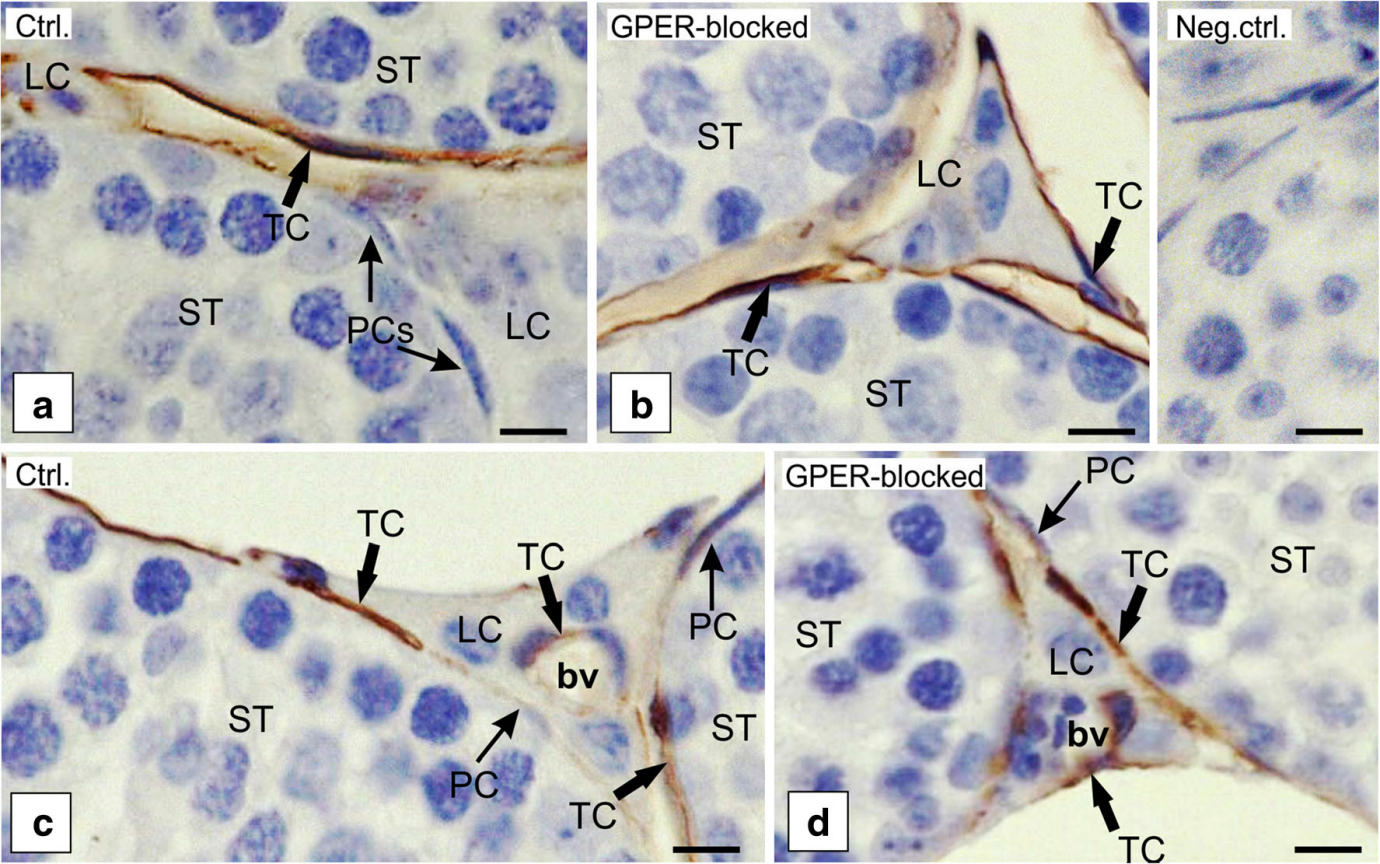
Presence of telocytes in mouse testis—immunohistochemical analysis. Effect of GPER blockage. Representative microphotographs of CD34 immunohistochemical localization in control (**a**, **c**) and GPER-blocked (**b**, **d**) mouse testes. Immunostaining with DAB and counterstaining with hematoxylin. Scale bars represent 15 μm. Immunoreaction was performed on testicular serial sections from at least three animals of each experimental group. Insert at **b**—negative controls. bv, blood vessels; LC, Leydig cells; PC, peritubular cells; ST, seminiferous tubules; TC, telocyte

**Fig. 4:**
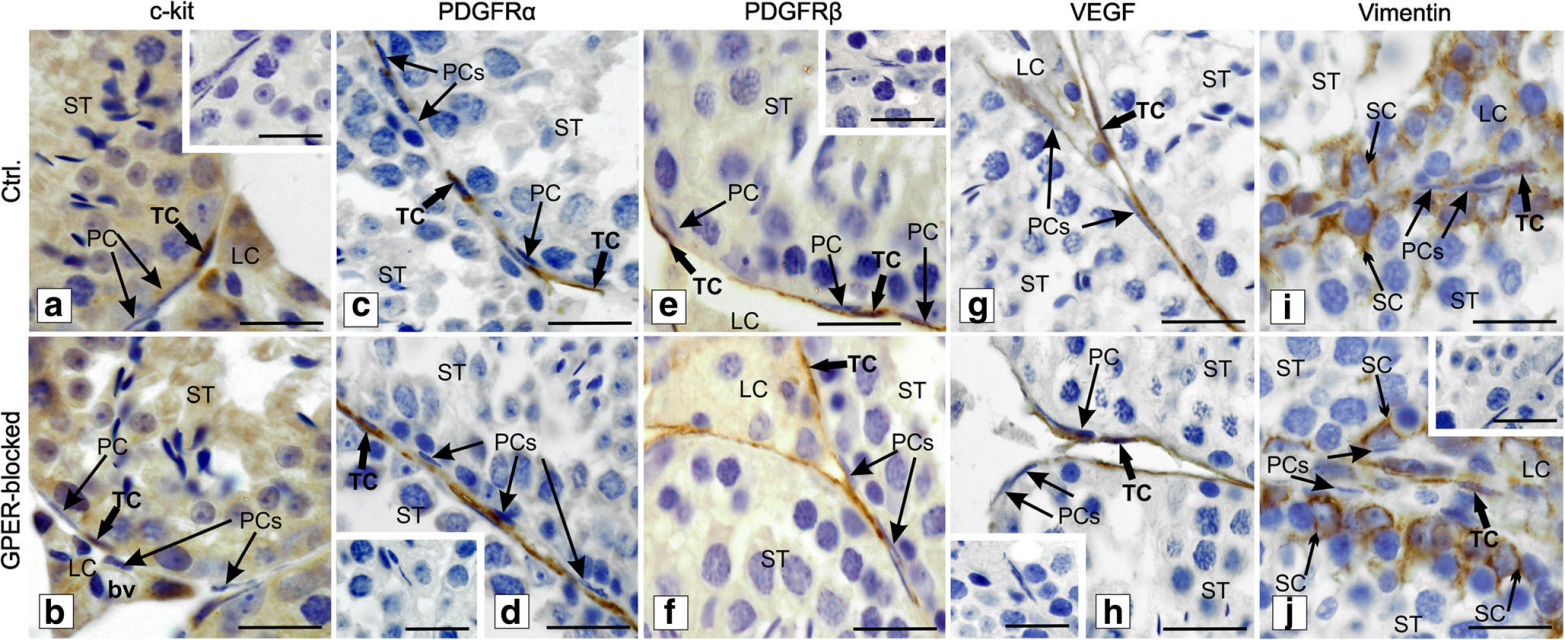
Presence of telocytes in mouse testis—immunohistochemical analysis. Effect of GPER blockage. Representative microphotographs of c-kit, PDGFRα and β, VEGF, and vimentin immunohistochemical localization in control (**a**, **c**, **e**, **g**, **i**) and GPER-blocked (**b**, **d**, **f**, **h**, **j**) mouse testes. Immunostaining with DAB and counterstaining with hematoxylin. Scale bars represent 15 μm. Immunoreaction was performed on testicular serial sections from at least three animals of each experimental group. Inserts at **a**, **d**, **e**, **h**, and **j**—negative controls. bv, blood vessels; LC, Leydig cells; PC, peritubular cells; SC, Sertoli cells; ST, seminiferous tubules; TC, telocyte

**Fig. 5:**
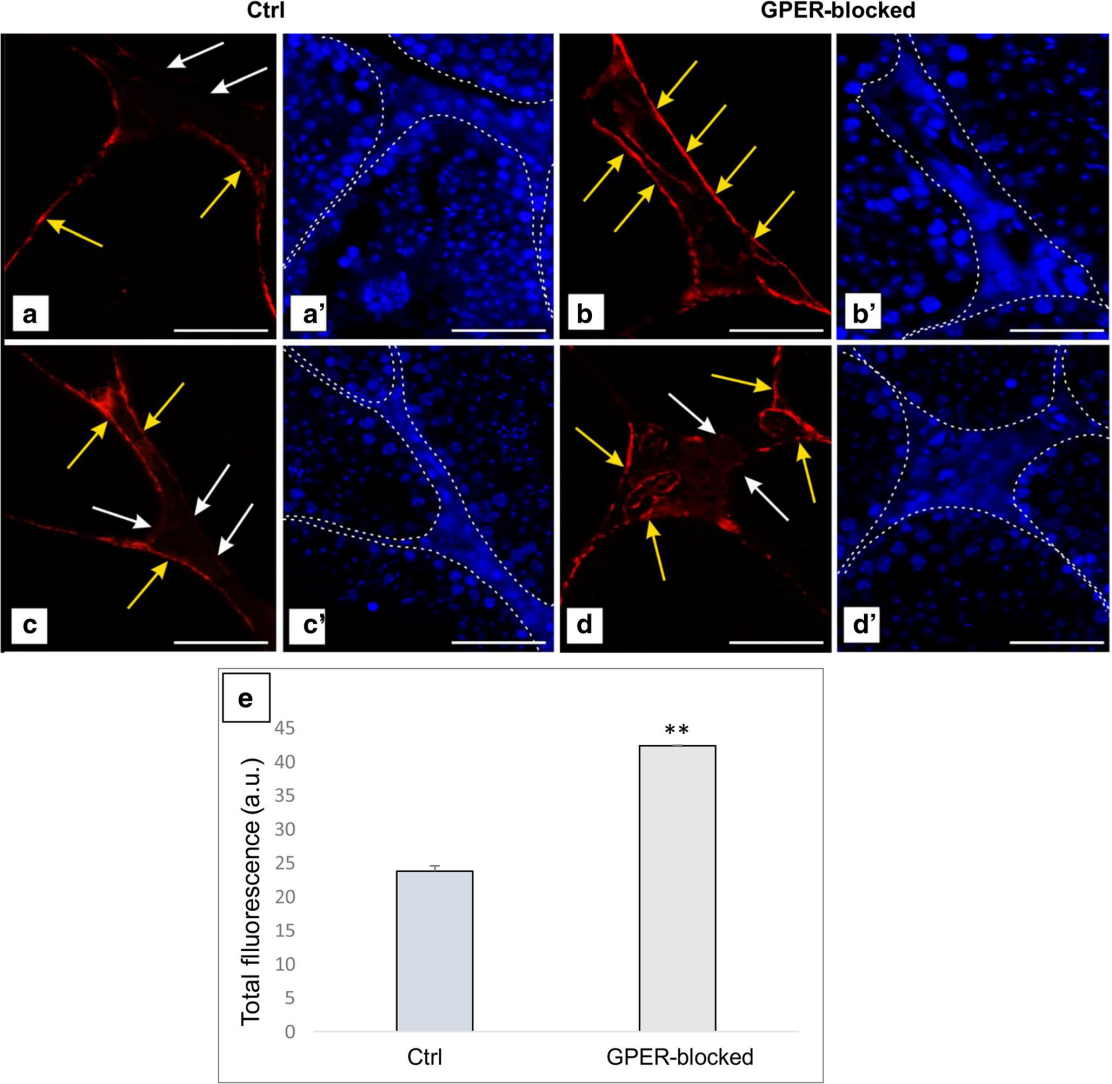
Presence of telocytes in mouse testis—fluorescence analysis. Effect of GPER blockage. Representative microphotographs of F-actin distribution in control and GPER-blocked mouse testes (A, A′, B, B′, C, C′, D, D′). Fluorescence with DAPI. Scale bars represent 20 μm. Dashed lines mark the periphery of interstitial tissue. White arrows—positive stained telopodes; yellow arrows—places with lack of staining (lack of TCs and/or telopodes). Quantitative analysis of fluorescence (E). Histograms of fluorescent intensities expressed as relative fluorescence (arbitrary units; a.u.). Immunoreaction was performed on testicular serial sections from at least three animals of each experimental group. Data is expressed as means ± SD. Asterisks show significant differences between control and GPER-blocked testes. Values are denoted as ^**^p < 0.01

Light microscopic observations were undertaken as an attempt for the identification and confirmation of TC location based on immunohistochemical staining for CD34 as well as c-kit, PDGFRα, PDGFRβ, VEGF, vimentin, and F-actin (Figs. 3, 4, and 5). Telocytes were located in between peritubular cells and surrounded groups of Leydig cells (Figs. 3 and 4). Moreover, single TCs positive for CD34 were identified between pericytes of blood vessels (Fig. 3c, d). Of note, no staining for CD34 was seen in other types of testicular cells, pericytes and peritubular cells, Leydig cells, and cells of the seminiferous tubules (Fig. 3). For PDGFRα, PDGFβ, and VEGF, positive staining was not seen in Leydig cells and cells of seminiferous tubules (Fig. 4c–h). On the contrary, staining for c-kit and vimentin revealed that not only TCs express these proteins but also spermatogenic cells (positive for c-kit) and Sertoli cells and peritubular cells (positive for vimentin) (Fig. 4a, b and i, j). Moreover, nonspecific staining for c-kit was detected in Leydig cells as well. Telocytes were observed in both control and GPER-blocked testes, but as the other types of testicular cells expressed c-kit, PDGFRα, PDGFRβ, VEGF, and vimentin, only CD34-positive cells were used for further analyses.

Telocytes that positively stained for CD34 were observed in both control and GPER-blocked mouse testes. In control testis, the number of TCs positive for CD34 was 9.4 ± 1.7 cells/testicular section, while it increased (19.1 ± 0.4** cells/testicular section) in GPER-blocked samples.

No staining was found in testicular sections incubated without primary antibody (inserts at Figs. 3b and 4a, d, e, h, j).

Long telopodes that stained strongly for F-actin were revealed in both control and GPER-blocked testis. Telopodes were lying in between peritubular cells and surrounded the interstitial space and blood vessels (Fig. 5). Discontinuous strong signal for F-actin (indicated on the presence of other cell types, e.g., peritubular-myoid in the peritubular area, pericytes in blood vessel epithelium and Leydig cells, fibroblast in the interstitium) when compared to DAPI staining was detected in the area outside seminiferous tubules. The relative fluorescence of F-actin was increased (*p* < 0.01) in comparison to controls (Fig. 5E).

### Expression of CD34, c-kit, PDGFRα, PDGFRβ, VEGF, and vimentin in mouse testis: effect of GPER blockage

**Fig. 6:**
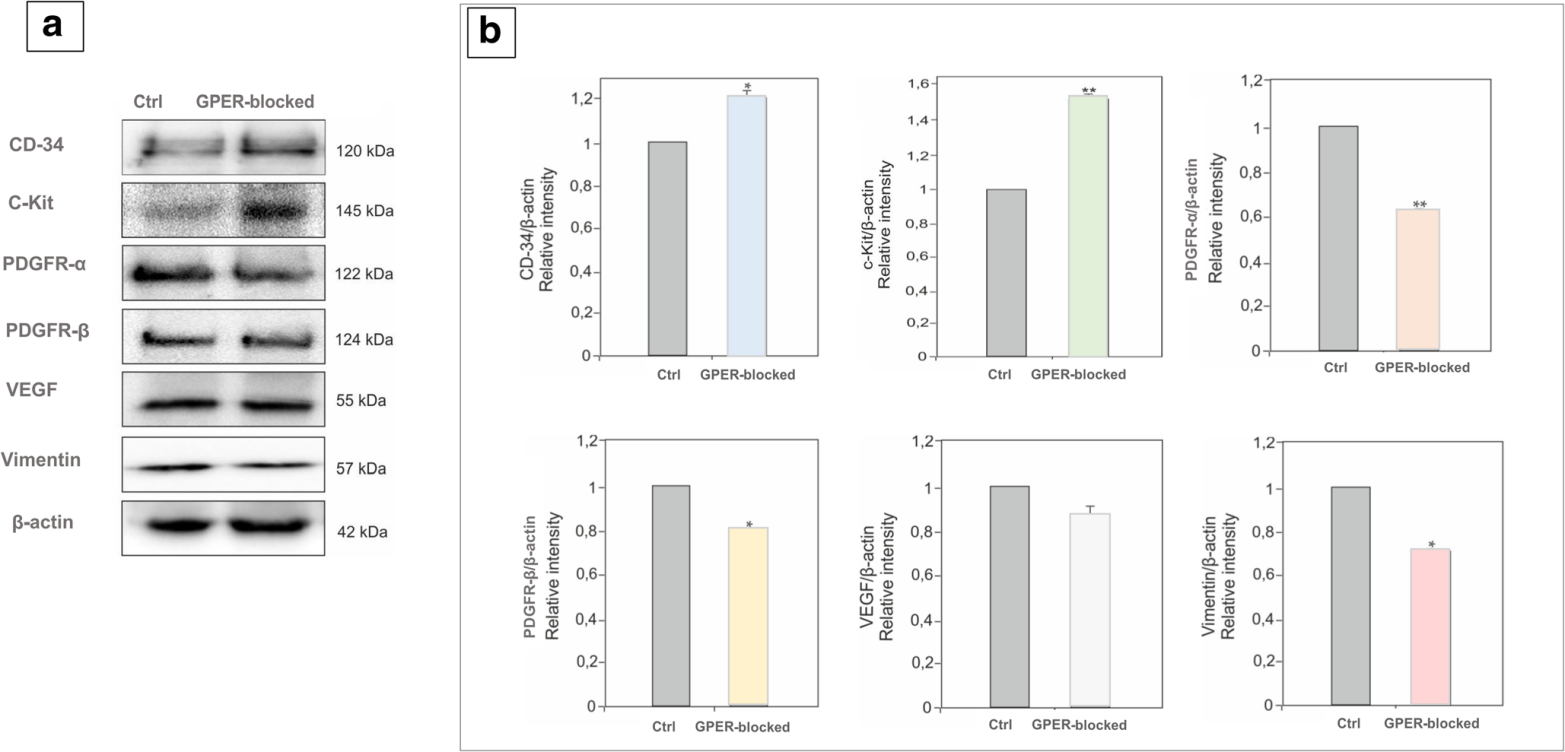
Expression of CD34, c-kit, PDGFRα, PDGFRβ, VEGF, and vimentin in mouse testis. Effect of GPER blockage. Representative blots of qualitative expression (**a**) and relative expression (arbitrary units) (**b**) of proteins CD34, c-kit, PDGFRα, PDGFRβ, VEGF, and vimentin in control and GPER-blocked mouse testes. Protein densitometry results are present below the corresponding blots. The relative amount of respective proteins normalized to β-actin. ROD from three separate analyses is expressed as means. From each animal, at least three samples were measured. Asterisks show significant differences control and GPER-blocked testes. Data is expressed as means. Values are denoted as ^∗^*p* < 0.05 and ^∗∗^*p* < 0.01

Changes in the level of telocyte marker proteins CD34, c-kit, PDGFRα, PDGFRβ, VEGF, and vimentin were found in G-15 testis when compared to the control (Fig. 6a, b). The protein level of CD34 was increased (*p* < 0.05) in GPER-blocked testis. The expression of c-kit was found to be increased (*p* < 0.01) too, while PDGFRα, PDGFRβ, VEGF, and vimentin expression decreased in GPER-blocked testis (*p* < 0.01; *p* < 0.05). Expression of VEGF was decreased but not significantly.

### Expression of CD34 and ERR mRNA in mouse testis: effect of GPER blockage

**Fig. 7:**
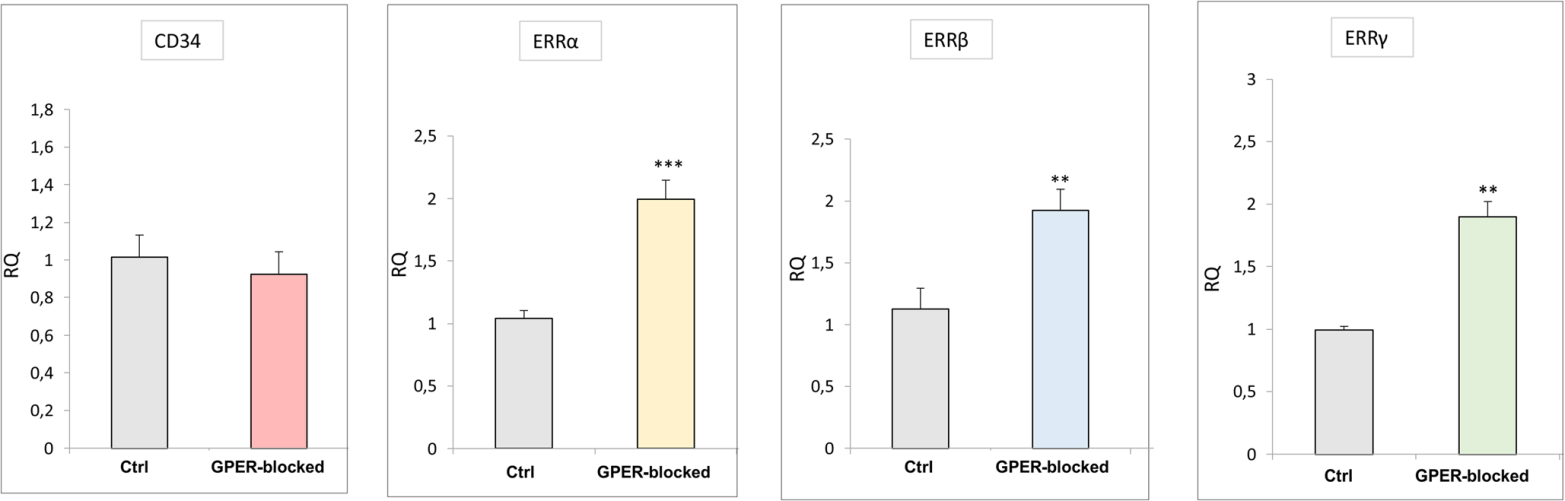
Expression of CD34 and ERRs mRNA in mouse testis. Effect of GPER blockage. Relative level (relative quantification; RQ) of mRNA for CD34, ERRα, ERRβ, and ERRγ in control and GPER-blocked mouse testes determined using real-time RT-PCR analysis 2^−ΔΔCt^ method. As an intrinsic control, β-actin mRNA level was measured in the samples. From each animal, at least three samples were measured. RQ is expressed as means ± SD. Asterisks show significant differences between control and GPER-blocked testes. Values are denoted as ^∗∗^*p* < 0.01 and ^∗∗∗^*p* < 0.001

No changes in CD34 mRNA levels were found in GPER-blocked testis in comparison to controls (Fig. 7). Alternatively, the mRNA expression of ERRα, β, and γ markedly increased (*p* < 0.01; *p* < 0.001).

### Intratesticular relaxin and Ca^2+^ concentrations: effect of GPER blockage

**Fig. 8:**
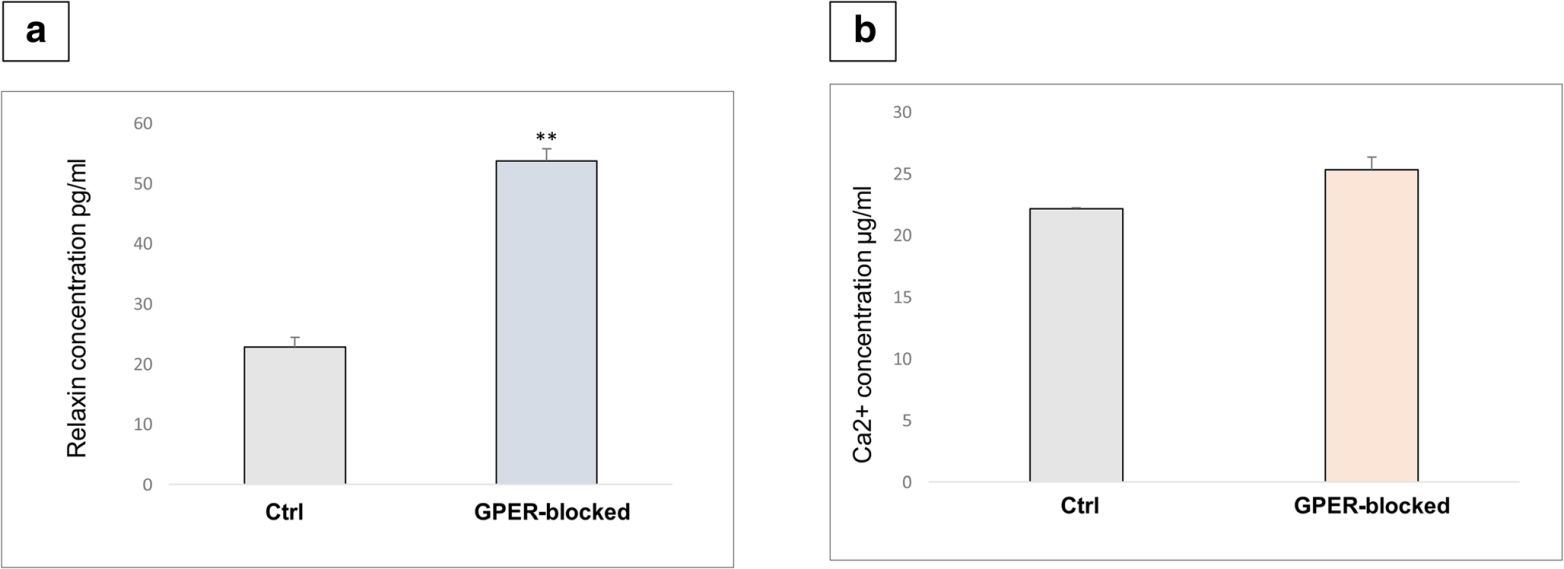
Intratesticular relaxin and Ca^2+^ concentrations. Effect of GPER blockage. Relaxin (**a**) and Ca^2+^ (**b**) concentration in control and GPER-blocked mouse testes. Data is expressed as means ± SD. From each animal, at least three samples were measured. Asterisks show significant differences between control and GPER-blocked testes. Values are denoted as ^∗∗^*p* < 0.01

A significant increase in relaxin concentration (*p* < 0.01) (Fig. 8a) and a slight increase in Ca^2+^ concentration (Fig. 8b) were revealed in GPER-blocked testis when compared to the control.

## Discussion

We report, for the first time, the presence of TCs in the interstitium, including the peritubular area, of mouse testis. Our results are in accord with the observations of Rodríguez et al. (2008) and Hasirci et al. (2017) on TC location in human testis. Telocytes reside in both peritubular and perivascular areas of the testis’ interstitium regardless of species (Yang et al. 2015). These cells are universally considered “connecting cells,” primarily involved in intercellular signaling. Telocytes have “strategic” positioning in a tissue, in between blood capillaries and their specific target cells (Popescu and Faussone-Pellegrini 2010) that in the testes can be especially cells outside seminiferous tubules. They are implicated in the electrical modulation of excitable tissue (the smooth muscle of the gut and uterus) and are capable of spontaneous initiation of electrical activity involving Ca^2+^ transients (Yamashita 2010). Moreover, TCs express ERα and the progesterone receptor, both of which are important hormone sensors (for review, see Roatesi et al. 2015).

When GPER was blocked in mouse testis, intratesticular estrogen levels, as well as estrogen feedback regulation of its own signaling, were altered (Kotula-Balak et al. 2018). Potential differences in the number of TCs of the control and GPER-blocked testes could exist as we revealed interstitial tissue-marked histological modulations for the first time. It is possible that TCs as well as other cells of the peritubular and interstitial compartments, peritubular-myoid cells, and Leydig cells may express GPER (Sandner et al. 2014; Zarzycka et al. 2016; Kotula-Balak et al. 2018). Therefore, changes in TC number may be a response to perturbed estrogen signaling and/or are a result of modulated function of neighboring cells as was reported in physiological and pathological conditions of various human organs (Cretoiu et al. 2012; Milia et al. 2013; Wang et al. 2014; Fu et al. 2015; Xiao et al. 2016).

As a first step, herein, general morphological characteristics of interstitial cells were assessed by SEM. Testis tissue is composed of two compartments made up of various cell types. Therefore, some limitations of SEM should be mentioned. Firstly, cutting testicular tissue requires precision and further processing needs to be gentle so as not to destroy the seminiferous tubules, as those cells can contaminate the interstitial space. Second, for analysis, only tissue which is spontaneously and exactly broken (during the procedure) in-between tubules allows for observation of the interstitial compartment.

Ultrastructural observations revealed that testicular TCs have remarkably long*,* thin, and moniliform, actin-rich cellular projections referred to as telopodes. Of note, for identification of telopodes of testicular TCs, F-actin seems to be an accurate and helpful marker, that clearly distinguishes F-actin-rich structures from the ones equipped with scarce microfilaments and/or arranged in a different way (present in other testicular cell types) when using basic fluorescence microscopy. The same morphological features were described for TCs in other tissues (Nicolescu et al. 2012; Cretoiu et al. 2012; Milia et al. 2013; Li et al. 2014; Rosa et al. 2018). In peritubular-myoid cells, abundant actin filaments are distributed in a species- and tissue-specific manner. In rats, the filaments within one peritubular cell run both longitudinally and circularly to the long axis of the seminiferous tubule, exhibiting a lattice-work pattern (Maekawa et al. 1996). In capillary cross sections, circumferential pericytes showed numerous parallel bundles of actin filaments forming a cap over the adjacent endothelial cells with a few actin filaments only (Wallow and Burnside 1980). Telocytes, peritubular cells, pericytes, and other cells of the interstitium may act in a coordinated manner to control contractility (via both cytoskeleton components including F-actin microfilaments and mitochondrial energy) of the interstitium, seminiferous tubules, and vessels, as well as modulate properties of the interstitial microenvironment.

As a second step, according to Popescu and Faussone-Pellegrini (2010) after electron microscopic TC identification, we tried to find the most suitable protein marker for identification of testicular TCs. Depending on tissue and species studied, diverse TC markers were identified through many years (Popescu and Faussone-Pellegrini 2010). From the mesenchymal cell markers commonly used for TC identification, e.g., CD34, c-kit, PDGFRα and β, VEGF, and vimentin, two of them c-kit and vimentin seem to be not suitable enough for distinguishing testicular TCs from other testicular mesenchymal cells, e.g., pericytes, fibroblasts as well as other types of testicular cells, e.g., Leydig cells, and macrophages (Feng et al. 1999; Fu et al. 2015; Zhou et al. 2015; Xiao et al. 2016). In addition, especially for c-kit, nonspecific staining occurred, too. On the other hand, CD34 seems to be the most relevant/helpful one; however, it is still not perfect when studying TCs in the testis.

Based on our results, changes in the expression of CD34, c-kit, PDGFRα and β, VEGF, and vimentin showed that either TC number and protein expression or number and protein expression of other interstitial cells can be GPER-dependent. Of note, differences in the intensity of staining between individual protein analyzed by immunohistochemistry and Western blot can be related to different tissue preparations, e.g., fixation, blocking of nonspecific staining defined for each analyses, and thus, specific epitope antibody recognition. A significant increase in GPER expression in cells surrounding seminiferous tubules was found in men with mixed atrophy, although detailed description of cell type and number was not provided (Sandner et al. 2014). Also, increased TC number was reported by Hasirci et al. (2017) in the testis of men with maturation arrest and Sertoli cell-only syndrome. The authors also suggested that TCs act as pacemaker cells that serve to induce spermatogenesis. Similarly, TC content is crucial for the stimulation of prostate function (McHale et al. 2006). In contrast, in patients with testicular atrophy and fibrosis, the number of TCs was reduced due to deformation of the testicular tissue.

A series of studies have revealed that sex steroid imbalance, caused by either hormonal or nonhormonal endogenous and exogenous factors, is responsible for changes in quantity and function of testicular cells (Schanbacher et al. 1987; Abney and Myers 1991; Hejmej et al. 2005; Gould et al. 2007; Carreau and Hess 2010; Lucas et al. 2011; Kotula-Balak et al. 2012; Rebourcet et al. 2014; Soliman and Emeish 2017). Moreover, in endocrine tissues, receptor number is controlled via hormone levels. Expression changes in one type of estrogen receptor affect the function of other estrogen receptors in various tissues and physiological conditions (Balasinor et al. 2010; Nephew et al. 2000; Kang et al. 2010; Madeira et al. 2013; Naugle et al. 2014; Boscia et al. 2015; Trejter et al. 2015; Kotula-Balak et al. 2018, b). In this study, mRNA expression of CD34 varied along with that of ERR; however, their expression trended in opposite directions. This indicates the influence of TCs on the testis interstitium and/or reversely on TCs via GPER and ERR signaling. Transcription and translation can be differentially controlled as is reflected here for CD34 mRNA and protein expression. In addition, the half-life of protein can be increased while its degradation is reduced in GPER-blocked testes.

Based on our previous results, Leydig cell ultrastructure following GPER blockage was characterized by lipid droplets surrounded via concentrically in structure endoplasmic reticulum but also degenerating (combined with a lipophagy) lipid droplets (Kotula-Balak et al. 2018). In the present study, no changes in TC ultrastructure in the control and GPER-blocked testis were revealed. Such a result reflects the higher sensitivity of Leydig cells to changes in hormonal interstitium microenvironment than seen in TCs. Also, TC structure and undiscovered function was not based on high-energy metabolism when compared to Leydig cells. We found that the absence of GPER does not induce perturbation of TC function at the organelle level.

Alterations in estrogen signaling and cellular communication following GPER blockage, along with tendency to number changes, can lead to further histological alterations of the interstitial tissue, e.g., hypertrophy or fibrosis (Haines et al. 2012), for example, via TC functional alterations and/or effect of these alterations on functionality of other interstitial cells.

In GPER-blocked testis, increased relaxin concentrations, exclusively secreted by interstitial cells (e.g., Leydig cells), indicate potential tissue histological changes. Indeed, we have lastly demonstrated the association of estrogen, ERR, and relaxin in bank vole interstitium overgrowth (Pawlicki et al. 2017). Possible tissue remodeling, early malignant transformation, or fibrosis (due to alterations mainly in the function of fibroblasts) should not be excluded. The development of relaxin-null mice provided particularly strong evidence that relaxin functions to protect against fibrosis (Samuel et al. 2007; Bennett 2009). The role of canonical estrogen receptors and estradiol in the development of cardiac, renal, and systemic fibrosis was also evidenced (Pedram et al. 2010; Hewitson et al. 2012; Aida-Yasuoka et al. 2013). Notably, loss of TCs accompanies fibrosis of multiple organs in systemic sclerosis (Manetti et al. 2014). In Caucasians, cystic fibrosis is linked to infertility (Sokol 2001).

In GPER-blocked testis, modulation of estrogen signaling affected TC distribution, potentially TC number and probably TC function, reflecting changes in the tissue’s histological appearance. In the light of these data, interaction of TCs, including possibly the secretory one, with estrogen and relaxin signaling supports TC involvement in interstitial tissue architecture and function. In addition, through TC release of inflammatory factors such as cytokines and interferons, their involvement in local immuno-inflammatory processes is feasible (Li et al. 2014; Ye et al. 2017).

According to Fu et al. (2015) and Ibba-Manneschi et al. (2016), enhancing the growth and/or survival of TCs could be an additional antifibrotic therapeutic strategy in many organs. Nowadays, in clinical andrology, treatment solutions for precocious gonad aging and tumorigenesis are intensively seeking (Giwercman and Giwercman 2011). Based on our results, GPER and ERR signaling modulation should be considered in future studies regarding the use of TCs against tissue pathological changes.

As mentioned above, TCs communicate via paracrine hormones but also via gap junctions that can be closed in response to high concentrations of Ca^2+^ (for review, see Calì et al. 2015). The heart rate is increased by relaxin modulation of the Ca^2+^ current in cardiac pacemaker cells (Han et al. 1994). In TCs of the female reproductive system, T-type Ca^2+^ channels contribute to the mechanical sensing of TCs, and what is more, estradiol controls its voltage gate (Banciu et al. 2018; Cretoiu et al. 2015). Interestingly, in isolated rat uterus, relaxin plays a double role as a transporter and buffer of Ca^2+^ (Fields 2005). For contraction of the rat testicular capsule, Ca^2+^ is needed (da Silva Júnior et al. 2013). Hence, Ca^2+^, together with relaxin, of which the contractile properties are well-known, is an important player controlling the interstitium tonus and creating the interstitial microenvironment. Our studies revealed no marked changes in Ca^2+^ level in GPER-blocked testis; thus, GPER is not directly implicated in Ca^2+^ regulation and it is possible that testicular TCs are not directly implicated in Ca^2+^ signaling. Future studies are warranted to elucidate the potential role of lipid droplets in TCs and their lipid homeostasis regulation apparently not by Ca^2+^.

Based on our current observations (direct lines of evidence from electron microscopic studies and indirect from immuohistochemical studies), we report, for the first time, the presence of TCs in mouse testis together with practical information regarding the analysis of TCs in electron microscopy and light microscopy (via protein markers) that can be useful for identification of testicular TCs. We hypothesize TC implication through tendency in their number changes in contractile and secretory function and/or their regulation of other interstitial cells in estrogen microenvironment including GPER-ERR interaction. Further studies in order to develop specific methods for TC identification and isolation and studies of their molecular characteristics and role in the testis are needed.
